# Coding-Complete Genome Sequences of Two SARS-CoV-2 Isolates from Egypt

**DOI:** 10.1128/MRA.00489-20

**Published:** 2020-05-28

**Authors:** Ahmed Kandeil, Ahmed Mostafa, Rabeh El-Shesheny, Mahmoud Shehata, Wael H. Roshdy, Shymaa Showky Ahmed, Mokhtar Gomaa, Ahmed El Taweel, Ahmed E. Kayed, Sara H. Mahmoud, Yassmin Moatasim, Omnia Kutkat, Mina Nabil Kamel, Noura Mahrous, Mohamed El Sayes, Nancy M. El Guindy, Amal Naguib, Mohamed A. Ali

**Affiliations:** a Center of Scientiﬁc Excellence for Inﬂuenza Viruses, National Research Centre, Giza, Egypt; b Central Public Health Laboratory, Ministry of Health and Population, Cairo, Egypt; Queens College

## Abstract

This report announces the complete genome sequences of two severe acute respiratory syndrome coronavirus 2 (SARS-CoV-2) isolates detected in Egypt. The isolates were obtained from oropharyngeal swab specimens from two Egyptians in Upper and Lower Egypt. Sequence analysis showed mutations that differentiate Egyptian strains from the reference strain 2019-nCoV WHU01.

## ANNOUNCEMENT

Coronaviruses (CoVs) are genetically differentiated into four genera, alpha-, beta-, gamma-, and delta-CoVs, within the family *Coronaviridae* (subfamily *Coronavirinae*) of the *Nidovirales* order. The beta-CoVs are further classified into four lineages (A to D) (1).

On 13 March 2020, 33 passengers and 12 staff members on a Nile cruise ship traveling between Luxor and Aswan tested positive for COVID-19. This unprecedented outbreak prompted the Egyptian government on 14 March to announce tougher prevention measures to control the expansion of COVID-19. As of 27 April 2020, Egypt had confirmed 4,782 cases in total, including 337 deaths (case-fatality rate, 7%).

Here, we announce the genome sequences of two severe acute respiratory syndrome coronavirus 2 (SARS-CoV2) isolates detected in two Egyptian patients. The isolate hCoV-19/Egypt/NRC-1/2020 is from an oropharyngeal swab specimen that was collected on 18 March 2020 from a 10-year-old boy from Damietta Governorate (P1). His father and brother concurrently tested positive for COVID-19. This family cluster of COVID-19 occurred coincidentally after the family attended the funeral of a person with a fatal Egyptian case of COVID-19 who died in Italy and was transferred for burial in Egypt. Available data excluded direct contact of the infected members of this family cluster with the corpse. Nevertheless, there was direct contact by hands and cheeks with the deceased man’s family, who may have directly contacted the corpse, which is a common funeral practice in Egypt. The second isolate, hCoV-19/Egypt/NRC-3/2020, was collected on 18 March as an oropharyngeal swab specimen from a 34-year-old woman from El-Minya Governorate (P2). This case was in contact with an infected relative in the group of the Nile River cruise outbreak at Luxor.

Viral RNA was extracted using a QIAmp viral RNA minikit (Qiagen, Germany). Reverse transcription was performed using a Superscript III system (Life Technologies) with random hexamers. Multiplex PCR was conducted using Q5 high-fidelity DNA polymerase (New England Biolabs). PCR products were purified using an Illustra GFX PCR DNA and gel band purification kit (GE Healthcare). DNA libraries were prepared using Nextera XT DNA-Seq library prep kits (Illumina, San Diego, CA, USA) according to the manufacturer’s instructions. Pooled libraries were sequenced with an Illumina MiSeq personal genome sequencer with 150-bp paired-end reads. CLC Genomics Workbench version 20 (CLC Bio, Qiagen) was used to analyze and process the sequencing reads. Whole-genome comparison of the two isolates revealed >99.9% identity with four nucleotide mutations at sites 8658, 15907, 19906, and 18877. Whole-genome comparison of these Egyptian isolates revealed >99.8% identity with isolate 2019-nCoV WHU01 (GenBank accession number NC_045512). Five nucleotide mutations were recorded among the Egyptian isolates, comparable with the reference 2019-nCoV WHU01 strain mutations C241T, C3037T, C14408T, A23260G, and G25563T.

The SARS-CoV-2 genome sequence of P1 was related to that of a Taiwanese patient traveling to Dubai and Egypt in February (hCoV-19/Taiwan/NTU03/2020|EPI_ISL_413592) except at sites 8658, 15907, and 18877 with substitutions of A to G, G to A, and C to T, respectively.

The SARS-CoV-2 genome sequence of P2 was closely related to that of a Japanese passenger (hCoV-19/Japan/P3-1/2020|EPI_ISL_419299) on a Nile River cruise ship who was characterized as a positive case on 9 March 2020. Three specific mutations were recorded in the virus detected in P2 from Egypt, at sites T4278C, G18963T, and C26692 (numbering based on the WHU01 strain) compared to the sequence of the Japanese isolate.

By comparing the amino acid sequences of the OrF1ab gene of the two isolates, substitutions of K to R and G to S were observed at positions 2798 and 5214 in the sequence of the P1 isolate. A mutation, A6247S, was characterized in the OrF1ab gene of the P2 isolate compared with the ancestral WHU01 strain as well as the P1 virus. The spike proteins of both Egyptian strains are completely identical in that they have a G to D substitution at site 614 compared with the ancestral WHU01 strain. Similar to the ancestral WHU01 strain, an identical polybasic furin cleavage site, RRAR, is found at the junction between the two subunits of the spike protein. The six receptor-binding domain (RBD) residues, L455, F486, Q493, S494, N501, and Y505, which were characterized to be critical for binding to angiotensin-converting enzyme 2 (ACE2) receptors, are identical in the two Egyptian isolates and are congruent to the ancestral WHU01 strain (2). The two Egyptian strains have H instead of Q at site 57 of open reading frame 3a (ORF3a) compared with the ancestral WHU01 strain.

**FIG 1:**
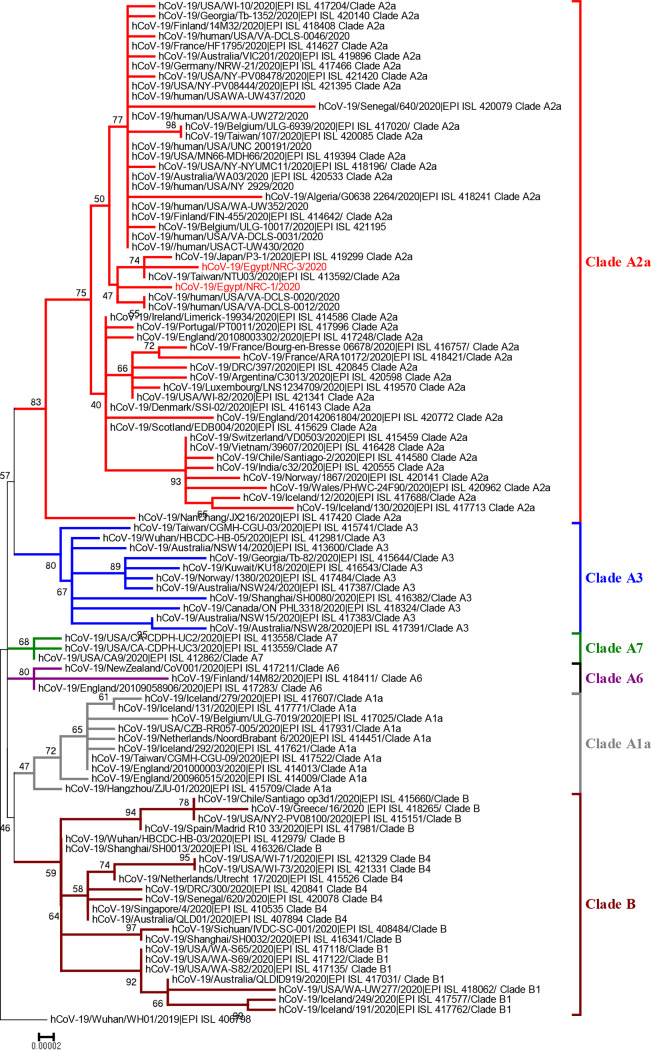
Neighbor-joining phylogenetic tree of a SARS-CoV-2 strain from Egypt and other global strains. The two Egyptian strains are shown in red. The percentage of replicate trees in which the associated taxa clustered together in the bootstrap test (1,000 replicates) is shown at the dendrogram nodes. The phylogenetic analysis was performed using MEGA7.

To further investigate the genetic relationship between Egyptian SARS-CoV-2 isolates and those of other available strains in the GISAID database, the nucleotide sequences of the Egyptian strains were aligned using BioEdit version 7.0.5.3 and ClustalW with SARS-CoV-2 strains from different countries. Using MEGA7 (3), a phylogenetic tree was constructed with the neighbor-joining method, and the reliability of each tree branch was estimated by performing 1,000 bootstrap replicates. Egyptian strains fell into clade A2a, which includes strains from Asia, Europe, the United States, Australia, and Africa (Fig. 1). The biological impact of the genetic and amino acid differences between the Egyptian SARS-CoV-2 isolates and the ancestral WHU01 strain on virus transmissibility, pathogenicity, and available therapeutic interventions demands rapid evaluation in further studies.

### Data availability.

These sequences have been deposited at the GISAID-EpiCoV newly emerging coronavirus SARS-CoV-2 platform under the identifiers EPI_ISL_430819 and EPI_ISL_430820 (available at https://www.gisaid.org/). The accession numbers for the Illumina MiSeq sequence raw reads in the NCBI Sequence Read Archive (SRA) are PRJNA629891 (BioProject), SAMN14814606 (BioSample, hCoV-19/Egypt/NRC-01/2020), SAMN14814607 (BioSample, hCoV-19/Egypt/NRC-03/2020), SRR11667146 (SRA, hCoV-19/Egypt/NRC-01/2020), and SRR11667145 (SRA, hCoV-19/Egypt/NRC-03/2020).
